# The impact of opioid-free labor epidural analgesia maternal and infant outcomes: a retrospective cohort study

**DOI:** 10.1016/j.bjane.2026.844750

**Published:** 2026-04-10

**Authors:** Kush S. Brahmbhatt, Ankith P. Reddy, Hiram A. Acevedo Bonilla, Ibrahim Tahashilder, Mohamed Ibrahim, Michelle Simon, Rakesh B. Vadhera, Rovnat Babazade

**Affiliations:** a The University of Texas Medical Branch at Galveston, John Sealy School of Medicine, Galveston, Texas; b University of Florida College of Medicine ‒ Jacksonville, Department of Anesthesiology, Jacksonville, Florida; c The University of Texas Medical Branch, Department of Biostatistics and Data Science, Galveston, Texas; d The University of Texas Medical Branch, Department of Anesthesiology, Galveston, Texas

**Keywords:** Analgesia, Epidural, Infant, Mothers, Opioid epidemic, Pregnancy outcome

## Abstract

**Background:**

Opioid Epidural Labor Analgesia (OLEA) is commonly used during labor. However, opioid use has been associated with adverse effects on maternal and fetal outcomes. Medications administered during epidural analgesia are systemically absorbed; therefore, we performed a retrospective cohort study to investigate whether Opioid-Free Labor Epidural Analgesia (OFLEA) is comparable to OLEA regarding maternal and infant outcomes at delivery.

**Methods:**

Of 1,423 patients initially identified, we excluded those with twin deliveries, duplicate records, or incomplete data. We then matched 1:1 on the mother’s age, including 618 patients for final data analysis. Our OLFEA group included an epidural solution of 0.2% ropivacaine, while our OLEA group included an epidural solution of 0.1% ropivacaine + 250 mcg fentanyl. Wilcoxon rank-sum tests were performed to assess our primary outcome of time-weighted pain scores before and after epidural placement. Secondary outcomes included duration (minutes) of maternal hypotension, tachycardia, and bradycardia episodes during labor, and incidence of neonatal fever, C-section, and Apgar scores (1 and 5 mins after delivery).

**Results:**

There was no significant difference between the OLEA and OFLEA groups in the time-weighted pain scores during labor before epidural placement. However, the time-weighted pain score was significantly lower in the OFLEA group (1.05 ± 1.52) compared to OLEA (1.43 ± 1.77, p = 0.006). Similarly, maximum pain scores after epidural were lower in OFLEA (3.32 ± 3.23) vs. OLEA (3.87 ± 3.33, p = 0.03). There were no significant differences in maternal hemodynamic events, Apgar scores, neonatal fever, or cesarean delivery rates.

**Conclusions:**

OFLEA is a safe and feasible alternative to OLEA. Avoidance of opioids may support safer maternal and neonatal care in obstetric anesthesia.

## Introduction

Each year, there are approximately 140 million births globally.^1^ In the United States, Opioid Epidural Labor Analgesia (OLEA) is used in most births.^2^ The burgeoning interest in opioid-free labor epidural analgesia stems from the escalating need to identify non-inferior alternatives to traditional opioid-based methods. This interest is driven by the growing recognition of the potential risks associated with even a single use of opioids during labor, which includes the possibility of Neonatal Opioid Withdrawal Syndrome (NOWS), maternal respiratory depression, and the initiation of long-term opioid dependence.^3^, ^4^, ^5^

In addition, epidural opioids adversely affect mothers before and after delivery (sedation, hypoxemia, hypotension, prolonged labor, itching, nausea and/or vomiting, infants' health, and prolonged hospital stay).^6^, ^7^, ^8^ Avoiding opioids in epidural solutions is a unique approach because most of it is systemically absorbed and is not necessarily better than giving systemic opioids. Despite this emerging problem, mother and baby are exposed to around 600 mcg of intravenous fentanyl equivalent dose during normal vaginal delivery.^9^ It would be clinically impactful if opioid-to-opioid-free epidural analgesia is as effective as continuous opioid epidural labor analgesia.

We, therefore, tested three main aims regarding OFLEA performance. Our primary aim was to determine if OFLEA is as effective as OLEA regarding labor pain management. We hypothesized that OFLEA is non-inferior to OLEA in regards to time weight average and highest pain scores during labor. Our second aim was to determine whether continuous OFLEA reduces the duration of hypotension (Mean Arterial Pressure [MAP] < 65 mmHg) and/or tachycardia (> 90 beats.min^-1^) and/or desaturation (< 90%) compared to OLEA. We hypothesized that the duration of hypotension, tachycardia, and desaturation would decrease in the OFLEA cohort. Lastly, our third aim was to study the effect of OFLEA on infant outcomes (Apgar scores and if required O_2_, intensive care unit admission) and maternal delivery outcomes (fever and incidence of cesarean section). We hypothesized that infant outcomes would improve in the OFLEA cohort. Our primary study hypothesis was that OFLEA is non-inferior to OLEA regarding these three aims.

## Methods

### Data source

This retrospective cohort study intended to study outcome differences in OFLEA and OLEA in labor pain management, intraoperative events, and infant outcomes. The protocols used were developed by authors at the University of Texas Medical Branch Department of Anesthesiology. The study was reviewed and given approval by the Institutional Review Board (IRB# 22-0149, 21-Jun-2022), and written informed consent was waived by the IRB. All data utilized in this study is from patients enrolled at UTMB between the dates of December 1, 2021, and June 9, 2022. This study adheres to the applicable STROBE guidelines.

### Cohort selection and development

The two experimental cohorts received epidural solutions of either continuous 0.1% ropivacaine + 250 mcg fentanyl (OLEA) or solely 0.2% ropivacaine (OFLEA). All parturients in our study received continuous epidural infusion (10‒13 mL.hr^-1^) with Patient-Controlled Epidural Analgesia (PCEA) enabled (4 mL bolus, 15-minute lockout). The only difference between groups was the epidural solution: the OFLEA group received 0.2% ropivacaine, whereas the OLEA group received 0.1% ropivacaine with 250 mcg fentanyl added to the epidural infusion bag. No initial bolus was administered; analgesia was provided solely through continuous infusion. All parturients requesting epidural analgesia for delivery at UTMB Health between December 1, 2021, and June 9, 2022.

**Figure 1:**
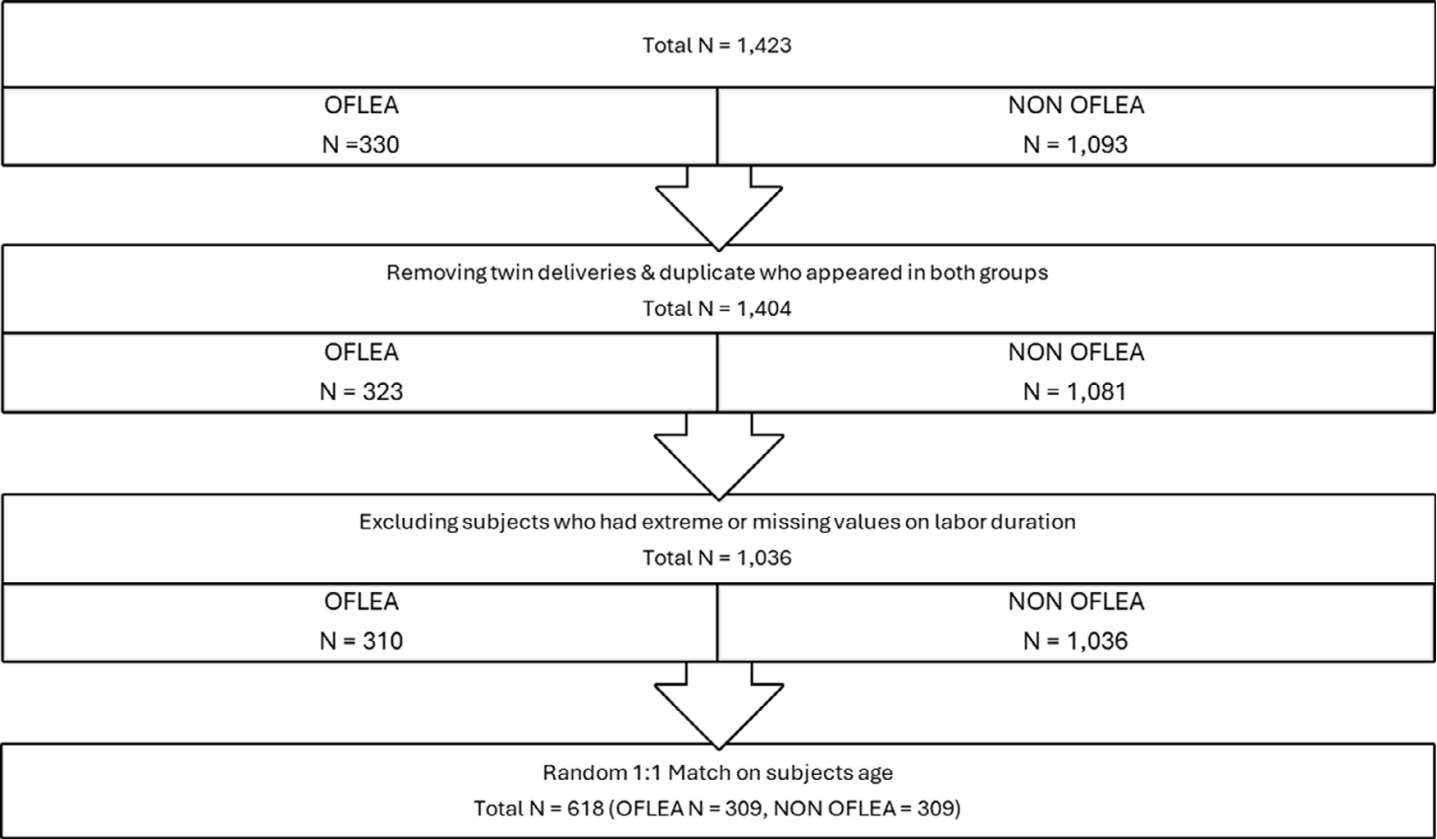
Flow diagram.

Participants were identified using the Epic Electronic Medical Record to stratify for an initial n = 1423 (OFLEA n = 330, OLEA n = 1093). We included pregnant women aged 18‒50 years of any parity and race who delivered under epidural spinal anesthesia during hospital admission. Those whose diagnostic criteria had not been clearly documented or established, patients of gestational age before 24 weeks who presented to the labor and delivery unit, and those who did not receive epidural anesthesia during hospital admission were excluded from the study. Patient characteristics including age, mode of delivery, and mode of anesthesia were obtained from chart review. After removing patients having twin deliveries that were duplicated in either group or who had extraneous amounts of missing values, we arrived at a total n = 1346 (OFLEA n = 310, OLEA n = 1036). Matching (1:1) on the mother’s age gave a final total of n = 618 (OFLEA/OLEA n = 309) (Fig. 1).

### Statistical analysis

For Aim 1, a non-parametric, Wilcoxon rank-sum test (Mann-Whitney *U*), average time-weighted pain scores and max pain scores during labor across OFLEA and OLEA groups were used. For Aim 2, we also utilized a non-parametric Wilcoxon rank-sum test to assess the total duration of episodes of tachycardia (> 90 beats.min^-1^), hypotension (< 65 mmHg, MAP), and hypoxemia (< 90% O_2_ sat) during labor. For Aim 3, the newborn’s outcome Apgar score equality was also checked with a non-parametric, Wilcoxon rank-sum test. If specific markers were found statistically significant, a linear regression model was created for the outcome. We found time-weighted pain score during labor after epidural and max pain score were significant and hence followed by multiple linear regression model adjusted for age, ethnicity, and fever, selected based on their potential to confound the relationship between treatment and pain outcomes.

## Results

A total of 618 age-matched parturient were included in the final data analysis. The OFLEA and OLEA groups consisted of 309 patients. The mean age in both groups was 26.64 years (SD = 5.59); 59.87% of participants identified as Hispanic (n = 370) and 45.31% of patients experienced fever during labor (n = 280) (Table 1); 80.74% (n = 499) of patients delivered via normal spontaneous vaginal birth, 12.94% (n = 80) had a cesarean delivery, 3.24% (n = 20) had a vaginal, vacuum assisted delivery, as well as 1 breech and 1 forceps assisted deliveries.

**Table 1:** Demographics of study participants.

Demographics	Mean (n)	SD (%)
Age (Mean, SD)	26.64	5.59
**Fever**		
No	338	54.69%
Yes	280	45.31%
BMI (Mean, SD)	33.44	6.34
Weight (Mean, SD), kg	86.37	18.47
**ASA Class**		
2	520	84.14%
3	98	15.86%
Gestational Age (Mean, SD), day	268.6	14.73
Gestational Age (Mean, SD), week	38.37	2.1
**Ethnicity**		
Hispanic	370	59.87%
Not Hispanic	248	40.13%
**Delivery Type**		
Normal Spontaneous Vaginal Birth	499	80.74%
C-Section, Low Transverse	80	12.94%
Vaginal, Vacuum (Extractor)	20	3.24%
Vaginal Birth after Cesarean Section	15	2.43%
Other (See Comments)	2	0.32%
Vaginal, Breech	1	0.16%
Vaginal, Forceps	1	0.16%

### Time-weighted and max pain scores after epidural placement

There was no significant difference between the two groups (OLEA and OFLEA) in the time-weighted pain score during labor before epidural placement. The time-weighted pain score (mean ± SD) before epidural for the OLEA group was 2.268 ± 2.217 vs. 2.617 ± 2.063 in the OLFEA group (p = 0.15). The time-weighted pain score during labor after epidural placement in the OFLEA group was statistically significantly lower than the OLEA group. The time-weighted pain score (mean ± SD) after epidural for the OLEA group was 1.430 ± 1.77 vs. 1.054 ± 1.517 in the OLFEA group (p = 0.0057). The parameter estimates for the linear model for the time-weighted pain scored during labor after epidural was -0.413 (95% CI -0.682, -0.144) (p = 0.0035). The max pain score during labor before epidural was not significantly different between groups. The max pain score (mean ± SD) before epidural for the OLEA group was 5.802 ± 2.647 vs. 5.756 ± 2.655 in the OFLEA group (p = 0.9058). The OFLEA group had a significantly lower max pain score during labor after epidural versus the OLEA group. The max pain score during labor after epidural for the OLEA group was 3.866 ± 3.325 vs. 3.318 ± 3.233 for the OFLEA group (p = 0.0345). The parameter estimates for the linear model for the max pain score during labor after epidural was -0.534 (95% CI -1.076, 0.0077) (p = 0.0881) (Table 2).

**Table 2:** Mean time weighted pain scores for OLEA and OFLEA.

Variables/Characteristics	OFLEA				p-value
	No		Yes		
	Mean (SD)	95% CI	Mean (SD)	95% CI	
Time-weighted pain score during labor before epidural	2.87 (2.22)	(2.62, 3.12)	2.62 (2.06)	(2.39, 2.85)	0.15
Time-weighted pain score during labor after epidural	1.43 (1.77)	(1.23, 1.63)	1.05 (1.52)	0.88 (1.23)	0.0057^a^
Max pain score during labor before epidural	5.8 (2.65)	(5.51, 6.1)	5.76 (2.65)	(5.46, 6.05)	0.9058
Max pain score during labor after epidural	3.87 (3.33)	(3.48, 4.25)	3.32 (3.23)	(2.95, 3.69)	0.0345^a^

**Outcome Variable**	**Intercept**	**R-square**	**OFLEA Estimate**	**95% CI**	**p-value**

Time-weighted pain score during labor after epidural	1.5	0.031	−0.413	(−0.682, −0.144)	0.0035^a^
Max pain score during labor after epidural	3.72	0.017	−0.534	(−1.076, 0.0077)	0.0881

**Sensitivity analysis with different margin for non-inferiority**					

**Margin (Δ)**	**Observed Difference**	**Distance from Margin**	**Power**		

1	−0.3763	0.6237	19.70%		
0.75	−0.3763	0.3737	92.30%		
1.5	−0.3763	1.1237	99.10%		

a Provides statistical significance at 0.05 level. Notes: p-values come from nonparametric Wilcoxon rank-sum tests since the data points do not follow normality assumptions. Each linear model was adjusted for age, ethnicity, and fever. Asterisk provides statistical significance at 0.05 level. For the time-weighted pain score during labor after epidural outcome, the p-value is significant. The parameter estimate described as using OFLEA will decrease 0.413 unit time-weighted pain score during labor after epidural.

### Hypotension, tachycardia, and bradycardia episodes during labor

The total duration (minutes) of tachycardia episodes during labor before epidural was not significantly different between groups. The total duration of tachycardia episodes during labor before epidural (mean ± SD) for the OLEA group was 200.04 ± 337.56 vs. 154.98 ± 296.64 for the OFLEA group (p = 0.2463). The median (IQR) total duration of tachycardia episodes during labor before epidural for the OLEA group was 54.50 (0 to 222.00) vs. 45 (0 to 167.00) for the OFLEA group (p = 0.2463). The total duration (minutes) of tachycardia episodes during labor after epidural was not significantly different between groups. The total duration of tachycardia episodes during labor after epidural (mean ± SD) for the OLEA group was 132.85 ± 181.40 vs. 128.59 ± 226.10 for the OFLEA group (p = 0.0532). The median (IQR) total duration of tachycardia episodes during labor after epidural for the OLEA group was 61 (3.50 to 197.00) vs. 30 (0 to 150.00) for the OFLEA group (p = 0.0532) (Table 3).

**Table 3:** Mean duration of adverse events during OLEA and OFLEA labor.

Variables/Characteristics	OLEA		OFLEA		p-value
	Mean (SD)	95% CI	Mean (SD)	95% CI	
Total duration (minutes) of episodes of tachycardia during labor before epidural	200.04 (337.56)	(157.99, 242.09)	154.98 (296.64)	(118.39, 191.56)	0.2463
Total duration (minutes) of episodes of tachycardia during labor after epidural	132.85 (181.40)	(109.78, 155.92)	128.59 (226.10)	(100.81, 156.36)	0.0532
Total duration (minutes) of episodes of hypotension during labor before epidural using BP	2.57 (7.75)	(−0.33, 5.46)	4.58 (10.31)	(0.23, 8.94)	0.6252
Total duration (minutes) of episodes of hypotension during labor after epidural using BP	3.92 (10.87)	(1.40, 6.44)	6.66 (21.19)	(1.91, 11.40)	0.6724
Total duration (minutes) of episodes of bradycardia during labor before epidural	32.11 (68.17)	(11.39, 52.84)	23.17 (48.41)	(9.11, 37.22)	0.3296
Total duration (minutes) of episodes of bradycardia during labor after epidural	16.95 (40.87)	(3.7, 30.2)	31.74 (59.02)	(14.41, 49.07)	0.2388

Notes: p-values come from nonparametric Wilcoxon rank-sum tests since the data points do not follow normality assumptions. Since none of the p-values are statistically significant at a 0.05 level, we did not fit regression models. Since the standard deviations are quite high, we also reported the interquartile ranges (Q1 to Q3), and this range shows the middle 50% of values when ordered from lowest to highest. Value of Q1 can be explained as the value under which 25% of data points are found when they are arranged in increasing order. Value of Q3 can be explained as the value under which 75% of data points are found when they are arranged in increasing order.

The total duration (minutes) of hypotension episodes during labor before epidural was not significantly different between groups. The total duration of hypotension episodes during labor before epidural (mean ± SD) for the OLEA group was 2.57 ± 7.75 vs. 4.58 ± 10.31 for the OFLEA group (p = 0.6252). The median (IQR) total duration of hypotension episodes during labor before epidural for the OLEA group was 0.0 (0.0 to 0.0) vs. 0.0 (0.0 to 0.0) for the OFLEA group (p = 0.6252). The total duration (minutes) of hypotension episodes during labor after epidural was not significantly different between groups. The total duration of hypotension episodes during labor after epidural (mean ± SD) for the OLEA group was 3.92 ± 10.87 vs. 6.66 ± 21.19 for the OFLEA group (p = 0.6724). The median (IQR) total duration of hypotension episodes during labor after epidural for the OLEA group was 0.0 (0.0 to 2.0) vs. 0.0 (0.0 to 0.0) for the OFLEA group (p = 0.6724) (Table 3).

The total duration (minutes) of bradycardia episodes during labor before epidural was not significantly different between groups. The total duration of bradycardia episodes during labor before epidural (mean ± SD) for the OLEA group was 32.11 ± 68.17 vs. 23.17 ± 48.41 for the OFLEA group (p = 0.3296). The median (IQR) total duration of bradycardia episodes during labor before epidural for the OLEA group was 0.0 (0.0 to 30.0) vs. 0.0 (0.0 to 30.0) for the OFLEA group (p = 0.3296). The total duration (minutes) of bradycardia episodes during labor after epidural was not significantly different between groups. The total duration of bradycardia episodes during labor after epidural (mean ± SD) for the OLEA group was 16.95 ± 40.87 vs. 31.74 ± 59.02 for the OFLEA group (p = 0.2388). The median (IQR) total duration of bradycardia episodes during labor after epidural for the OLEA group was 0.0 (0.0 to 15.0) vs. 0.0 (0.0 to 30.0) for the OFLEA group (p = 0.2388) (Table 3).

### Incidence of maternal labor side effects

The number of patients who experienced at least 1 episode of tachycardia during labor before epidural for the OLEA group was 250 (80.91%) vs. 255 (82.52%) for the OFLEA group. The incidence, n (%), of patients that experienced at least 1 episode of tachycardia during labor after epidural for the OLEA group was 240 (77.67%) vs. 257 (83.17%) for the OFLEA group (Table 4).

**Table 4:** Number of maternal labor side effects OLEA and OFLEA.

Variables/Characteristics	OLEA	OFLEA
	n (%)	n (%)
At least 1-episode of tachycardia during labor before epidural	250 (80.91)	255 (82.52)
At least 1-episode of tachycardia during labor after epidural	240 (77.67)	257 (83.17)
At least 1-episode of hypotension during labor before epidural using BP	30 (9.71)	24 (7.77)
At least 1-episode of hypotension during labor after epidural using BP	74 (23.95)	79 (25.57)
At least 1-episode of bradycardia during labor before epidural	44 (14.24)	48 (15.53)
At least 1-episode of bradycardia during labor after epidural	39 (12.62)	47 (15.21)

Notes: This table shows the number and percentages of subjects who experienced at least one episode for each of the above characteristics during pre and post epidurals. For example, out of 309 ‘No OFLEA’ subjects, 250 (80.91%) subjects experienced at least 1 episode of tachycardia during labor before epidural and 255 (82.52%) ‘OFLEA yes’ subjects experienced at least one episode of tachycardia before epidural. All other variables/characteristics can be described in a similar way.

The number of patients that experienced at least 1 episode of hypotension during labor before epidural for the OLEA group was 30 (9.71%) vs. 24 (7.77%) for the OFLEA group. The incidence, n (%), of patients that experienced at least 1 episode of tachycardia during labor after epidural for the OLEA group was 74 (23.95%) vs. 79 (25.57%) for the OFLEA group (Table 4).

The number of patients that experienced at least 1 episode of bradycardia and during labor before epidural for the OLEA group was 44 (14.24%) vs. 24 (15.53%) for the OFLEA group. The incidence, n (%), of patients that experienced at least 1 episode of bradycardia during labor after epidural for the OLEA group was 39 (12.62%) vs. 47 (15.21%) for the OFLEA group (Table 4).

### Neonatal outcomes

There was no significant difference in Apgar scores for the infants both 1-minute and 5 minutes from delivery. The Apgar score for the infant 1-min from delivery (mean ± SD) for the OLEA group was 7.896 ± 1.29 vs. 7.819 ± 1.26 for the OFLEA group (p = 0.0892). The Apgar score for the infant 5 min from delivery (mean ± SD) for the OLEA group was 8.760 ± 1.16 vs. 8.743 ± 1.174 for the OFLEA group (p = 0.5784) (Table 5).

**Table 5:** Mean apgar scores for OLEA and OFLEA groups.

Variables/Characteristics	OFLEA				p-value
	No		Yes		
	Mean (SD)	95% CI	Mean (SD)	95% CI	
Apgar score for baby 1-min from delivery	7.89 (1.29)	(7.74, 8.05)	7.82 (1.26)	(7.67, 7.97)	0.0892
Apgar score for baby 5 min from delivery	8.76 (1.16)	(8.62, 8.89)	8.74 (1.17)	(8.6, 8.88)	0.5748

Notes: p-values come from nonparametric Wilcoxon rank-sum tests since the data points do not follow normality assumptions. As none of the p-values are statistically significant at the 0.05 level, we did not fit regression models.

There was also no significant difference in the incidence of fever in neonates between the OFLEA and OLEA groups; 141 (45.63%) neonates in the OLEA group developed fever after delivery compared to 139 (44.98%) in the OFLEA group (p = 0.872). In contrast, 168 neonates in the OLEA group did not develop fever compared to 170 (55.02%) neonates in the OFLEA group (Table 6).

**Table 6:** Fever and C-Section Incidence in OLEA and OFLEA groups.

Variables/Characteristics	Total	n (%)		p-value
		OLEA	OFLEA	
**Total**	618	309 (50)	309 (50)	
**Fever**				
No	338	168 (54.37)	170 (55.02)	0.872
Yes	280	141 (45.63)	139 (44.98)	
**C-section**				
No	524	262 (84.79)	262 (84.79)	1.000
Yes	94	47 (15.21)	47 (15.21)	

Notes: Percentages shown as column percentage. For example, variable ‘Fever’ percentages can be described as: among 309 No OFLEA subjects, 168 (54.37%) had fever and 141 (45.63%) had no fever. On the other hand, among 309 ‘OFLEA’ subjects, 170 (55.02%) had fever and 139 (44.98%) had no fever. C-Section generated from delivery type information. So, if the delivery type included ‘C-Section low transverse’ or ‘vaginal birth after cesarean section’ they were flagged as C-section. The p-values come from the Chi-Square test. As none of the p-values are significant at the 0.05 level, we can conclude that there is no association between fever and OFLEA (yes/no) C-section and OFLEA.

Lastly, no significant difference was identified in the incidence of C-section delivery between the OFLEA and OLEA groups; 47 patients (15.21%) underwent a C-section in both the OFLEA and OLEA groups, while 262 patients (84.79%) did not have a C-section in both the OFLEA and OLEA groups (p = 1.000) (Table 6).

## Discussion

Our retrospective cohort study studied the effect of OFLEA on pain reduction after epidural placement in 618 patients compared to OLEA. We found that OFLEA was non-inferior to OLEA after epidural placement in the reduction of pain scores. One meta-analysis identified similar findings of noninferiority of OFLEA both immediately and 24 hours postpartum.^10^ Multimodal analgesia has also been used to achieve similar pain-free responses; one study found a higher dose of propofol alone was needed to achieve loss of response compared to propofol with remifentanil.^11^ Our study used a similar approach of administering a higher concentration of ropivacaine in our OFLEA group. Studies further investigating the use of multimodal labor analgesia regimens are necessary to identify avenues to improve pain control.

The OFLEA group in our study used a higher concentration of ropivacaine compared to the OLEA group. A study comparing postoperative pain after epidural analgesia using 0.5% ropivacaine compared to 0.75% ropivacaine and 0.5% bupivacaine found greater pain control with a higher concentration of local analgesia.^12^ Although this may not be generalizable to labor, it shows its efficacy as a modality of local analgesia. Another study using 0.0625% bupivacaine/0.0002% fentanyl compared to 0.125% bupivacaine identified no significant difference in pain control during any stage of labor.^13^ This dose-dependent effect of local analgesia may partly be explained by a faster onset of sensory and motor loss.^14^ In our study, a higher concentration of local analgesia in the OFLEA regimen may explain the noninferiority in pain control. Another benefit of using a higher local analgesic concentration can be attributed to lower side effect profiles in the OFLEA group. Opioid analgesia is associated with adverse effects that may contribute to pain, most notably opioid-induced hyperalgesia.^15^^,^^16^ This has been shown to contribute to greater levels of pain after labor despite its intended perioperative use.^16^ A meta-analysis of opioid anesthesia on postoperative hyperalgesia found that high doses of remifentanil were associated with small yet significant increases in pain levels postoperatively; however, other studies have demonstrated no change in pain levels in surgical patients with opioid anesthesia regimens.^17^^,^^18^

We also studied the effects of OFLEA on the duration of episodes of tachycardia, hypotension, and bradycardia before and after labor. In this study, OLEA was non-inferior compared to OFLEA after epidural placement in duration of these adverse effects. One meta-analysis found that in select surgeries, opioid-free regimens significantly reduced duration of tachycardia, hypotension, and bradycardia without compromising pain control; however, two studies found contrasting evidence, reporting increases in the duration of these episodes when using opioid-free analgesia; one study, in particular, found that a dexmedetomidine dose of 0.4 to 1.4 μg.kg^-1^.h^-1^ is associated with severe episodes of bradycardia compared to an opioid-only regimen. Bradycardia was the most significant adverse effect reported in these two studies, while there were no differences in rates of tachycardia and hypotension between OFLEA and OLEA.^19^, ^20^, ^21^ Possible explanations for this finding are medication-related and dose-dependent effect on adverse outcomes, as a high-dose unimodal opioid-free regimen of dexmedetomidine was used in Beloeil et. al.^20^ Our data showed no significant difference in these outcomes when using unimodal ropivacaine. Previous literature has shown that dose reduction can be achieved using multimodal analgesia compared to unimodal analgesia to reduce adverse effects without compromising pain control.^22^ Studies using these regimens compared to opioid-inclusive anesthesia are needed to explain further differences in adverse outcomes and potentially identify a dosage that balances adequate pain control with side effect profile optimization.

We lastly studied rates of neonatal Apgar scores, fever incidence, and rates of C-section between OFLEA and OLEA. Our study showed that OFLEA is non-inferior to OLEA in terms of neonatal outcomes after delivery. While literature regarding this data is limited, the existing literature has not found an association between epidural analgesia and neonatal outcomes; the common doses of OLEA show no significant effect on Apgar scores and C-section rates.^23^^,^^24^ One study did show that epidural analgesia is associated with slightly lower (but above 7) Apgar scores in neonates who were exposed to labor epidural analgesia compared to non-epidural analgesia.^25^ Our lack of differences in neonatal outcomes may be due to potential underpowering of the study. Larger, multi-centered randomized controlled trials would be beneficial to further delineate the impact of OFLEA on neonatal outcomes. Neonatal withdrawal syndrome may be associated with maternal opioid usage during pregnancy, but no study has identified this association in the intrapartum setting. Overall, our study findings concurrent with existing literature suggest that OFLEA may be a safer alternative for neonates.

### Limitations

Our study has several limitations. We performed a single-center retrospective cohort study, which limits our power and generalizability. We also did not use the total volume or dose (mL) of local anesthetic administered, which limits our ability to fully evaluate dose-dependent effects on analgesia and motor block. The higher concentration of ropivacaine used in the OFLEA group (0.2% vs. 0.1% in OLEA) may have influenced outcomes and represents a potential confounder. We also did not include maternal side effects such as pruritus, nausea, or vomiting. Further, patient records were not all homogenous which may contribute to the variability seen in some of the data, such as pain scores and duration of maternal adverse outcomes. Our study population is not fully representative of American or global populations. Our average age at delivery was similar to 2021 national statistics (26.64 vs. 27.3). A large portion of our study population was Hispanic (n = 370, 59.87%) compared to 24.2% of mothers in 2021. The majority of our patient population underwent spontaneous vaginal delivery (n = 499, 80.74%) while the 2021 national percentage was 65.7%. Our cesarean delivery rates were lower than national rates (n = 80, 12.94% vs. 32.1%). Our Vaginal Birth After Cesarean Delivery (VBAC) rates were lower than 2021 averages (n = 15, 2.43% vs. 13.5%).^26^ These differences reflect institutional practice patterns that may not be generalizable to other centers. Additionally, we matched groups only on maternal age and did not adjust for other important obstetric factors such as parity, body mass index, comorbidities, or labor duration. Residual confounding is therefore likely, and unmeasured differences between groups could have influenced the outcomes. In addition, motor block and ambulation were not assessed in our retrospective data. These outcomes are fundamental when comparing local anesthetic concentrations, as higher doses can impair maternal mobility and affect the course of labor. The inability to evaluate motor block or the ability to walk is therefore an important limitation of our analysis.

## Conclusions

Our study findings suggest that OFLEA can be used as a safer alternative to OLEA. OFLEA is non-inferior to OLEA regarding maternal peripartum pain scores, adverse effects, and neonatal outcomes. Future studies identifying differences between OFLEA and OLEA will help tease the differences between these two modalities of analgesia, as the avoidance of opioid analgesia is a potential future alternative to OLEA; prospective trials are warranted to establish safety and efficacy.

## AI assistance disclosure

We did not use any AI tools in the preparation of the manuscript or in the analysis.

## Data availability statement

The datasets generated and/or analyzed during the current study are available from the corresponding author upon reasonable request.

## Authors’ contributions

Kush Brahmbhatt: This author helped in the protocol development, data extraction, data analysis, and the writing of the manuscript.

Ankith Reddy: This author helped in the protocol development, data extraction, data analysis, and the writing of the manuscript.

Hiram Acevedo Bonilla: This author helped in the protocol development and review of the manuscript.

Ibrahim Tahashilder: This author helped in the data extraction and data analysis in the manuscript.

Mohamed Ibrahim: This author helped in the protocol development, experiment conduction, and review of the manuscript.

Michelle Simon: This author helped in the protocol development, experiment conduction, and review of the manuscript.

Rakesh Vadhera: This author helped in the protocol development, experiment conduction, and review of the manuscript.

Rovnat Babazade: This author helped in the protocol development, experiment conduction, guidance of data extraction and analysis, and review of the manuscript.

## Funding

Seed Grant in Women’s Health Research. This is an award provided by the UTMB Center for Interdisciplinary Research in Women’s Health (CIRWH).

## Conflicts of interest

The authors declare no conflicts of interest.
